# The pathway and characteristics of patients with non-specific symptoms of cancer: a systematic review

**DOI:** 10.1186/s12885-022-09535-y

**Published:** 2022-05-23

**Authors:** Ellen Jensen, Jette Kolding Kristensen, Rikke Tveden Bjerglund, Søren Paaske Johnsen, Janus Laust Thomsen

**Affiliations:** 1 Center for General Practice, Department of Clinical Medicine, Aalborg University, Aalborg, Denmark; 2 The Quality Unit for General Practice in the North Region of Denmark (Nord-KAP), Aalborg, Denmark; 3 Danish Center for Clinical Health Service Research, Department of Clinical Medicine, Aalborg University, Aalborg, Denmark

**Keywords:** Diagnostic center, Patients with non-specific symptoms of cancer, General practice, Gut feeling, Patient characteristics

## Abstract

**Background:**

Non-specific symptoms are  common and often sign of a non-serious disease. Because of this, patients with non-specific symptoms of cancer (NSSC) present a challenge for general practitioners (GP). Studies describing characteristics of patients with NSSC have been done after fast-track pathways were created to diagnose and treat patients with NSSC. This study reviews characteristics of patients with NSSC and their patient pathways.

**Materials and methods:**

Database searches of Embase, Cochrane, PubMed, Cinahl and Web of Science were performed. Search terms used were *cancer, patient pathway*, and *NSSC* with their synonyms. The flow diagram *Preferring Reporting Items for Systematic Review* was applied to the systematic search. The *Newcastle–Ottawa Assessment Scale (NOS)* was used to compare the quality of the included studies.

**Results:**

Twelve studies met the inclusion criterias. All studies were considered to be of high methodological quality.

*Patient Pathway:* 11–35% of patients were diagnosed with cancer. Median number of days through diagnostic process was 7–10.

*Patient Characteristics*: The most prevalent cancers included hematological-(14–30%), gastrointestinal-(13–23%) and lung cancers (13%). Rheumatological, musculoskeletal and gastrointestinal diseases were among the most common non-malignant diseases diagnosed. Weight loss, fatigue, pain and loss of appetite were the most common symptoms. Cardiovascular diseases, lung diseases, diabetes and previous diagnosed cancer were the most common comorbidities. Mean age of included patients was 60–72 years.

**Conclusion:**

Limited number of studies were found and they lacked sufficient heterogenic data to conduct a metaanalysis. Symptoms, diagnoses, age and gender were described with some heterogenic results. Further studies should be conducted to gather broader knowledge about patients with NSSC.

## Introduction

Patients with undiagnosed cancer often present with a variety of symptoms at their initial visits to general practices [1, 2]. Alarming symptoms such as a breast lump or a color change in a mole are often described but non-specific symptoms such as tiredness or weight loss often present as well [3, 4].

These non-specific symptoms are usually signs of non-serious or chronic diseases which challenges general practitioners (GP) who need to recognize suspected cancer [1, 5]. Intuition (also known as “gut feeling”) is an essential diagnostic tool for doctors to use for patients with non-specific symptoms, and GPs can use intuition when referring patients for further examination for possible cancer [6]. How intuition works has not been fully explained in the literature which means that unspecified symptoms represent clinical challenges. Specifically, patients with unspecific symptoms may be diagnosed with cancer at a later stage which results in higher mortality rates [1].

In Australia and several European countries (including the UK, the Netherlands, Spain and the Scandinavian countries), GPs may refer patients with suspected cancer to specialized departments at hospitals through an urgent referral cancer package pathway (CPP) that ensures a streamlined diagnostic process [7–11]. In Denmark, a CPP system with 28 organ-specific cancer packages was established in 2007. In 2012, Denmark became the first European country to open a non-organ specific CPP for patients with non-specific symptoms of cancer (NSSC) [12, 13]. GPs can activate this non-organ specific CPP and secure a fast diagnostic work-up for patients with NSSC without having to choose between the organ-specific CPPs.

When GPs choose an organ-specific department for patients with NSSC, diagnosis results may be delayed if examinations and tests are negative, and patients need a referral to another organ-specific setting. The non-organ specific CPP in Denmark are facilitated in Diagnostic Centres (DC) located at the hospitals. Similar centres have also been implemented in Norway and Sweden [10].

The UK has a two-week waiting system for suspected cancer patients; however, a CPP for patients with NSSC has not yet been established in the British system [9, 14]. A cross-sectional study from the UK published in January 2020 found that patients with NSSC had a longer diagnostic process and were more likely to be diagnosed in emergency departments compared with patients who had specific cancer symptoms. This points to the need for an alternative pathway for NSSC patients being considered in the UK [14]. There are preliminary results from studies in the UK showing that a multidisciplinary cancer diagnostic clinic for patients with NSSC would be an effective way to diagnose cancer [15].

A number of studies conducted in association with the opening of DC and CPPs for patients with NSSC have described population characteristics, mortality, and other factors which influence the pathway for the patients with NSSC [10, 12, 16, 17]. Nonetheless, a full overview of the socioeconomic, mental, and physical factors with regard to patients with NSSC is lacking.

When looking at cancer patients in general, socioeconomic characteristics have been shown to have an impact on patient pathways. This was vital knowledge when extracting information for this review. A review from the UK in 2005 by L.M. Woods et.al. found that several studies showed an association between socioeconomic status and cancer survival [18]. Other studies also found that socioeconomic factors have an impact on cancer survival. The Woods study observed that married people have a significantly better survival rate and that comorbidity, nutrition and seeking healthcare could have an impact on survival. The authors concluded that further studies on how socioeconomic position and cancer survival is associated with how patients seek and obtain access to the system is important [18].

Mental, social, and physical characteristics are equally important for extracting a picture of patients’ characteristics. This corresponds to WHO’s definition of health being “physical, social and mental wellbeing and not merely absence of disease” [19]. This systematic review will summarize the characteristics of patients with NSSC (including physical, mental and socioeconomic characteristics) as well as the patient pathway including how patients seek and obtain access to the health care system.

## Materials and methods

This review follows the “Preferred Reporting Items for Systematic Reviews and Meta-Analyses” (PRISMA) recommendations. The review was prospectively registered in the PROSPERO database at https://www.crd.york.ac.uk/PROSPERO and assigned nr CRD42019129303.

### Search strategy and study selection

Databases used to identify studies for this systematic review included PubMed, Cochrane, Embase, Web of Science and CINAHL. The search topics included cancer, patient pathway and non-specific symptoms. The three search topics were further divided into specific search terms that were used in all databases using following synthesis (“diagnostic cent*”[Text Word] OR “non specific symptom*”[Text Word] OR “non specific sign*”[Text Word] OR “nonspecific symptom*”[Text Word] OR “nonspecific sign*”[Text Word] OR “gut feeling*”[Text Word]) AND (“Early Diagnosis”[MeSH Terms] OR “suspicion*”[Text Word] OR “Referral and Consultation”[MeSH Terms] OR “urgent referral*”[Text Word] OR “patient pathway*”[Text Word] OR “refer*”[Text Word] OR “delay*”[Text Word] OR “Time Factors”[MeSH Terms] OR “diagnostic cent*”[Text Word] OR “risk”[Text Word] OR “critical pathway”[Text Word]) AND (“Neoplasms”[MeSH Terms] OR “cancer”[Text Word] OR “serious disease*”[Text Word] OR “serious ill*”[Text Word] OR “seriously ill*”[Text Word] OR “carcinoma*”[Text Word] OR “tumor*”[Text Word] OR “tumour*”[Text Word]).

**Fig. 1:**
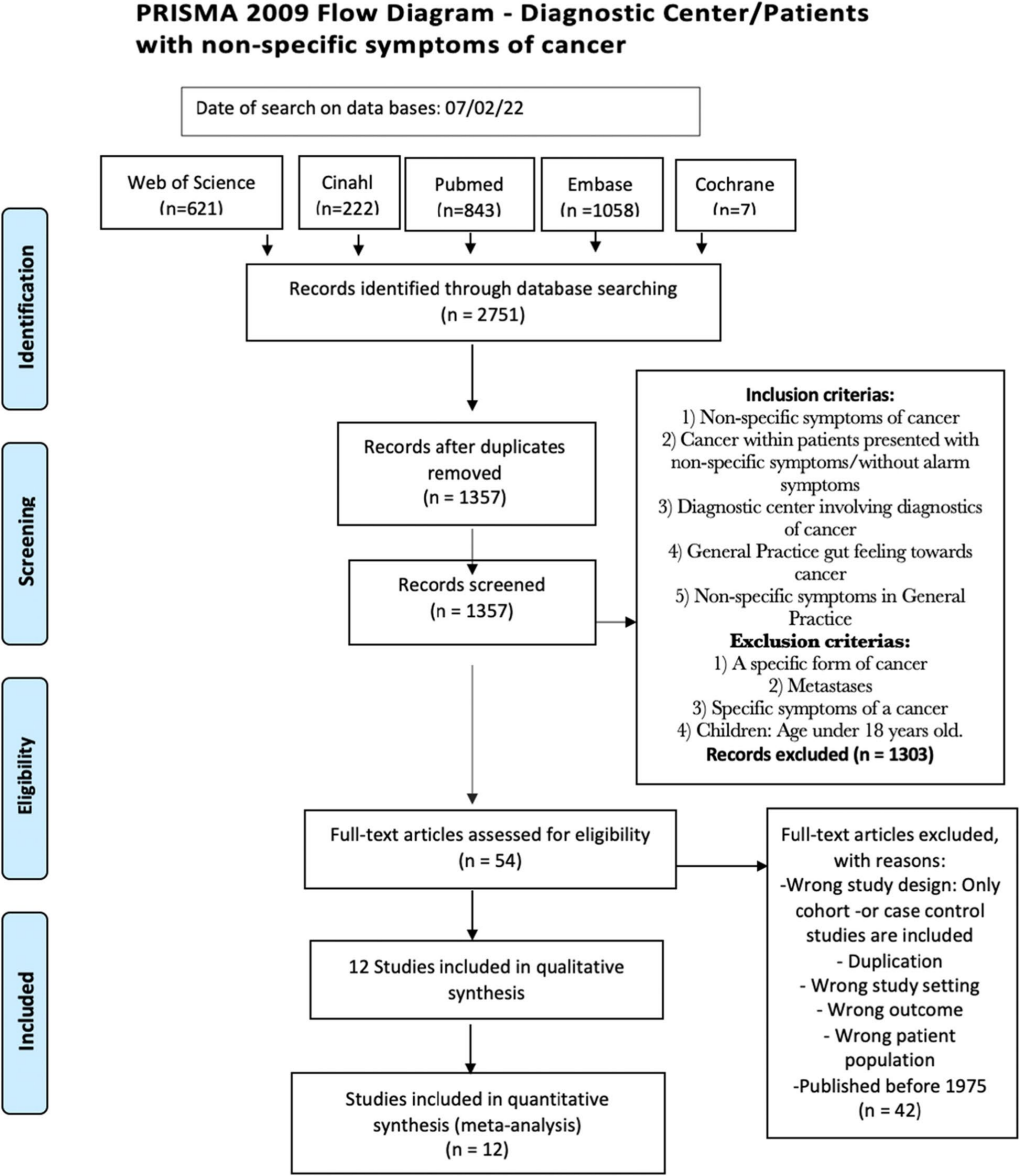
Prisma 2009 Flow Diagram-Diagnostic center/patients with non-specific symptoms of cancer

A flow diagram describing the identification, screening, eligibility and inclusion of studies is presented in Fig. 1. The process of extracting duplications was done through the reference management program *Covidence*. Abstract and full text screening was carried out through two reviewers (EJ, JLT and RTB). Disagreements were solved by discussion with a third party. Titles and abstracts were screened by using the inclusion and exclusion criteria listed in Table 1.

**Table 1:** Inclusion and exclusion criteria used for the “abstract and title” screening in this systematic review

	Inclusion	Exclusion
Criteria	1) Non-specific symptoms of cancer 2) Cancer within patients who presented with non-specific symptoms/without alarm symptoms 3) Diagnostic center involving diagnostics of cancer 4) General Practitioner’s gut feeling towards cancer 5) Non-specific symptoms in General Practice	1) A specific form of cancer 2) Metastases 3) Specific symptoms of cancer 4) Children: Age under 18 years old

All full text publications reporting cohort and case control studies were included, and all other types of studies were excluded.

The last update of the database search was attained January 2022. The restriction date for publications was set to 1 January 1975, and only publications written and published in English, Danish, Norwegian or Swedish. Denmark, Norway, Sweden and UK are the only countries who have an urgent referral pathway for patients with NSSC, hence it could be more likely that relevant studies have been made in the native languages. Studies from UK are pilot studies, hence urgent referral pathways and the related diagnostic center are not implemented in all of UK at this moment [20, 21]. Also authors language is Scandinavian hence these languages are naturally read.

We used the *Newcastle – Ottawa Quality Assessment Scale* to compare the methodological quality of the included studies [22] as shown in Table 2. There are eight items in the *Newcastle–Ottawa Quality Assessment Scale* and each item gives one point except for comparability which can give two points. This means the maximum points scored is nine. Scoring five points or under identifies studies with a high risk of bias [22].

**Table 2:** Quality assessment of included studies according to the Newcastle-Ottawa Quality Assessment Scale. Socioeconomic, physical and mental factors as well as patient pathway are additionally included in the schedule for

Assesment	Donker [23]	Jørgensen [12]	Moseholm [24]	Moseholm [25, 26]	Næser [16, 17]	Ingeman [27]	Stenman [10]	Dolly [21]	Møller [28]	Chapman [29]
**1. Representativeness of the exposed cohort**	+	+	+	+	+	+	+	+	+	+
**2. Selection of the non exposed cohort**	+	+	+	+	+	+	+	+	+	+
**3. Ascertainment of exposure**	+	+	+	+	+	+	+	+	+	+
**4. Demonstration that outcome of interest was not present at start of study**	+	+	+	+	+	+	+	+	+	+
**5. Comparability of cohorts on the basis of the design or analysis**	+	+	+	+	+	+	+	+	+	+
**6. Assessment of outcome**	+	+	+	+	+	+	+	+	+	+
**7. Was follow-up long enough for outcomes to occur**	+	+	+	+	+	+	+	+	+	+
**8. Adequacy of follow up of cohorts**	+	+	+	+	+	+	+	+	+	+
**Stars summarized**	8	8	8	8	8	8	8	8	8	8
**Quality: Low/medium/high**	High	High	High	High	High	High	High	High	High	High
**A. Socioeconomic characteristics**	+	+	+	+	+	+	+	+		
**B. Mental characteristics **	**–**	+	+	+	+	+	+	+		
**C. Physical characteristics**	+	+	+	+	+	+	+	+	+	
**D. Patient Pathway**	+	+	+	+	+	+	+	+	+	+

### Data extraction

Data on physical, mental and social patient characteristics were extracted from the studies. The patient pathway was also extracted. All data extracted is listed in Table 3 (Results) and divided into  the  categories; patient pathway, physical characteristics, social characteristics and mental characteristics. Data extraction is presented in Table 3.

**Table 3:** Results

Results										
Study										
	Donker [23]	Jørgensen [12]	Moseholm [24]	Moseholm [25, 26]	Næser [16, 17]	Ingeman [27]	Stenman [10]	Dolly [21]	Møller [28]	Chapman [29]
**Variables**										
**Patient Pathway**										
**% getting a cancer diagnose**	35	17	11	22	13	16	22	11		
**Cancer stage**							47% had solid tumors with potential to spread based on TNM-staging and 20% were referred to palliative care			Upper GI Stage 1: 4% Stage 2: 21% Stage 3: 17% Stage 4: 57% Lung Stage 1: 26% Stage 2: 9% Stage 3: 13% Stage 4: 53% Hematological Stage 1: 20% Stage 2: 13% Stage 3: 40% Stage 4: 27% Lower GI Stage 1: 0% Stage 17% Stage 3: 35% Stage 4: 48%
**Mortality**		Mortality 1-year rate at 44 and 3% for those with no cancer			The 1-year cumulative mortality is 28% compared to 3% for patients with another diagnose and 2% with no diagnose		Median survival time after cancer diagnosis was 1,4 years			
**Median referral from referral to last visit day**		Median time from first visitday at DC to last was 9 days	7	Duration of symptoms: 12 weeks	10	The median primary care interval for patients diagnosed with cancer was 15 days	From first contact in primary care to diagnosis at DC: Median time was 37 days investigation at DC: 77% were investigated within 22 days			
**Duration of symptoms before referred with NSSC**				Median duration of symptoms was 12 weeks				Without cancer: 2 weeks or less 1% 1 month or less 6% 1-3 month 19% 3–6 months 17% 6-12 months 14% With cancer: 2 weeks or less 1% 1 month or less 12% 1-3 month 33% 3–6 months 16% 6-12 months 15%		
**Laboratory tests**	44% patients had abnormal tests	Anemia, leukocytopenia, thrombocytopenia, elevated LDH and CRP was significant associated with cancer			A high-level lactate dehydrogenase had the strongest association with cancer	48% of patients had an abnormal blood sample at the GP, and 17% of patients that acquired a cancer diagnose had an abnormal cancer diagnose at the GP	59% of patients attaining a cancer diagnose were referred due to pathological lab values 61% of patients attaining a non-cancerous diagnose were referred due to pathological lab values 35% of patients attaining no diagnose were referred due to pathological lab values	(With cancer vs without cancer) Increased levels: Anaemia 43% vs 27%, Thrombocytosis 18% vs 6%, Raised CRP 68% vs 36% Liver dysfunction 18% vs 8%	Conclusion from the study: “Hit rate for detecting malignancy, in patients with non-specific symptoms and signs of cancer, seems comparable to other fast-track workup plans for patients with disease-specific symptoms”	
**Other paraclinical measures**	44% underwent advanced diagnostic imaging	77% underwent advanced diagnostic imaging	47% of the patients went through advanced imaging.		28% had a cancer diagnose when taking a supplementary CT scan	15% of patients had an abnormal diagnostic imaging, and 17% of patients that acquired a cancer diagnose had an abnormal diagnostic imaging				
**Physical Characteristics**										
**The 1. most prevalent cancer diagnoses**		Hematological 30%	Breast cancer 18%	Lymphoma 14%	Hematological 25%	Lung 18%	Hematological			Upper GI 22%
**The 2. most prevalent cancer diagnoses**		GI 23%	Hematopoietic/lymphoid tissue cancer 15%	Pulmonary cancer 13%	Colorectal 18%	Colorectal 13%	Lung			Lung 22%
**The 3. most prevalent cancer diagnoses**		Lung 10%	MM 12%	Colorectal 10%	Kidney 10%	Hematopoietic tissue cancer 10%	Colorectal			Hematological/lower GI, urological 13%
**The 1. most prevalent non-cancer diagnoses**		Rheumatological disease 12%	Cardiovascular disease 9%		Rheumatology 26%		Musculoskeletal diseases 20%			
**The 2. most prevalent non- cancer diagnoses**		GI 10%	Gastrointestinal disease 7%		Gastroenterology 18%		Gastrointestinal diseases 16%			
**The 3. most prevalent non-cancer diagnoses**		Hematological disease 9%	Musculoskeletal and connective tissue disease 5%		Endocrinology 15%		Hematological diseases 10%			
**Symptom nr. 1** **when referred with NSSC**	Weight loss 24%	Weight loss 39% of patients attaining a cancer diagnose and 42% of patients did not attaining a cancer diagnose		Weight loss 35%	Weight loss 53%	Weight loss 53% - Within patients with cancer 16%	Diagnosed with cancer: Pathological lab values 59% Diagnosed with other than cancer: Pathological lab values 61% No Diagnosis: Weight loss 48%	Fatigue Without cancer: 73% With cancer: 74%		Weightloss 66%
**Symptom nr. 2 when referred with NSSC**	Rare GP visits 22%	Fatigue 35% of patients attaining a cancer diagnose and 39% of patients did not attaining a cancer diagnose		Suspicion of major illness/cancer 15% and Pain 15%	Fatigue 50%	Fatigue 50% - Within patients with cancer 16%	Diagnosed with cancer: Weight loss 45% Diagnosed with other than cancer: Weight loss 38% No Diagnosis: Pathological lab values 35%	Pain Without cancer: 73% With cancer: 74%		GP clinical suspicion: 36%
**Symptom nr. 3 when referred with NSSC**	Duration of symptoms 19%	Loss of appetite 28% of patients attaining a cancer diagnose and 26% of patients did not attaining a cancer diagnose		Fatigue 13% and Abnormal blood tests 13%	Loss of appetite or nausea 36%	Pain 37% Within patients with cancer 18%	Diagnosed with cancer: Pain/joint pain 36% Diagnosed with other than cancer: Fatigue 35% No Diagnosis: Fatigue 33%	Weight loss Without cancer: 47% With cancer:: 37%		Pain 32%
**Comorbidity**		46% had no comorbidity, 46% had 1–2 comorbidity and 7% had 3 or more comorbidities	78% had no comorbidity, 19% had 1 comorbidity and 3% had 2 or more comorbidities	47% had no comorbidity, 31% had 1 comorbidity and 22% had 2 or more comorbidities	51% had no comorbidity, 16% had 1 comorbidity, 16% had 2 and 17% had 3 or more comorbidities	25% had no comorbidity, 34% had 1 comorbidity, 24% had 2 and 18% had 3 or more comorbidities		Without cancer: 1–2: 32% 3–5: 42% > 5: 14% With cancer: 1–2: 31% 3–5: 42% More than 5: 15%		31% had 0 comorbidities, 42% 1, 11% 2/3, 18% 3 and 9% comorbidity.
**The 1. most prevalent comorbidity in patients referred with NSSC**		Cardiovascular diseases: 16% of patients referred with NSSC	Cardiovascular diseases: 6% of patients referred with NSSC		Hypertension 23% of patients referred with NSSC	Hypertension 28% of patients referred with NSSC				
**The 2. most prevalent comorbidity in patients before referred with NSSC**		Lung diseases 13% of patients referred with NSSC. (10% of patients that acquired a cancer diagnose.)	Shared 2. place: Diabetes, Cerebrovascular disease, previous cancer: each 3% of patients referred with NSSC		Osteoarthritis or inflammatory arthritis: 12% of patients referred with NSSC	Chronic lung disease: 17% of patients referred with NSSC				
**The 3. most prevalent comorbidity in patients before referred with NSSC**		Previous diagnosed cancer: 12% of patients referred with NSSC	COPD: 2% of patients referred with NSSC		Earlier cancer or non melanoma skin cancer: 11,0% of patients referred with NSSC	Diabetes: 12% of patients referred with NSSC				
**The 1. most prevalent comorbidity of patients when diagnosed with cancer**		Previous diagnosed cancer: 22% of patients that acquired a cancer diagnose	Previous diagnosed cancer: 5% of patients that acquired a cancer diagnose							
**The 2. most prevalent comorbidity of patients diagnosed with cancer**		Cerebrovascular diseases: 12% of patients that acquired a cancer diagnose. (11% of patients referred with NSSC)	Cardiovascular diseases: 3% of patients that acquired a cancer diagnose							
**The 3. most prevalent comorbidity of patients diagnosed with cancer**		Cardiovascular diseases: 12% of patients that acquired a cancer diagnose.	Diabetes: 2% of patients that acquired a cancer diagnose							
**Other comorbidities examined**		Patients referred with NSSC: -Inflammatory diseases 12%. 7% of patients diagnosed with cancer -Diabetes 12%. 9% of patients diagnosed with cancer -Cerebrovascular diseases 11%. 12% of patients diagnosed with cancer -Renal failure 4%. 2% of patients diagnosed with cancer -Peptic ulcer 3%. 4% of patients diagnosed with cancer - Dementia 2%. 0% of patients diagnosed with cancer -Cirrhosis 0,4%. 0,7% of patients diagnosed with cancer	Patients referred with NSSC: -Connective tissue disease 1%. 0,5% of patients diagnosed with cancer		Patients referred with NSSC: -COPD 11% -Diabetes: 9% -Ischemic heart disease: 8% -Mental illness: 7% -Stroke 6% -Osteoporosis 6%	Patients referred with NSSC: -Ischemic heart disease 11% -Chronic joint or rheumatic disease 11% -Osteoporosis 6% -Apoplexy 5% -Moderate to severe mental disorder 5%				
**Mean age** **Of patients referred with NSSC**		67	65	64	70	66	69 Median age diagnosed with cancer: 70	Without: cancer: 62 With cancer: 69	Median age Malignancy not suspected: 68 Malignancy possible: 72	Median: 69 Mean: 67
**Age**	A year increase in age will ﻿increase the odds with a factor 1.02 for being diagnosed with cancer	Significantly associated to a cancer diagnose with an 34% increase in odds every ten-year	In 1 year increase in age, the odds will increase 4% for a cancer diagnose					The cancer patients had a statistically significantly higher age than those without cancer		
**Gender**	48% of referred patients were women and 52% men	47% of referred patients were women and 53% men 44% of patients diagnosed with cancer were women and 56% men 48% of patients diagnosed with a non-cancerous disease were women and 52% were men	Being woman was significant associated with being diagnosed with cancer 47% of referred patients were women and 53% men 58% of patients diagnosed with cancer were women and 42% men. 53% of patients diagnosed with a non-cancerous disease were women and 47% were men	53% of referred patients were women and 47% men	55% of referred patients were women and 45% men	53% of referred patients were women and 47% men Cancer were more often found in men than in women	51% of referred patients were women and 49% men	Without cancer: 39% males, 61% females With cancer: 60% males 40%females This was statistical significant.	Malignancy not suspected: Female 52% Malignancy possible: Female 55%	44% Male and 56% Female were referred with non-specific symptoms of cancer
**Social characteristics**										
**Marital status**			54% of the population was married patients. 56% of patients diagnosed with cancer were married	67% of the population was married, 12% separated, 11% widowed, 9% single	59% of the population of patients diagnosed with cancer was married		68% of patients referred with NSSC were married/living together and 31% were single			
Educational level*			50% of the study population had a medium academic education. 52% diagnosed with cancer had a medium education. 6% had a high academic education. 6% diagnosed with cancer had a high education. 41% had a short education/skilled worker. 39% diagnosed with cancer had a short education/skilled worker.	16% of the study population had a longer academic education and 28% a medium academic education. 34% had a short less than 15 years education/skilled worker	47% of patients diagnosed with cancer had a medium level education, 36% none education and 14% with a higher educational level.		46% with a low education level (</= 9 years) were referred with NSSC 36% with a medium education level (10-12 years) were referred with NSSC 18% with a higher education (> 12 years) were referred with NSSC			
**Occupation**			Patients referred with NSSC: 28% were employed. 68% were retired. 1% were out of workforce. Patients diagnosed with cancer: 18% were employed. Diagnose. 80% were retired. 1% were out of workforce.	Patients referred with NSS 37% were employed, 59% retired and 7% unemployed	17% of patients with cancer was working patients and 83% were retired. 0% was out of workforce.					
**Income**					27% of patients that acquired a cancer diagnose were in the lowest income, 54% in the middle income and 19% in the highest income. 29% of patients that did not acquire a non-malignant disease were in the lowest income, 48% in the middle income and 23% in the highest income					
**Smoking**	36% of 11 smokers included in study 1 had a cancer diagnose	34% of patients never smoked and 66% was former/current smokers. 30% diagnosed with cancer never smoked and 70% was former/current smokers		70% of the patients included in the study were former/current smokers and 25% never smoked	29% are current smokers and ﻿32% former/intermittent smoker and ﻿38% never smoked			Without cancer Current: 20% Former: 11% Never: 46% With cancer: Current: 26% Former: 18% Never: 56		
**Alcohol**		10% had a weekly consumption of alcohol above national guidelines. 13% diagnosed with cancer had a weekly consumption of alcohol above national guidelines			81% did not consume alcohol on daily basis			Without cancer Within recommendation limits 21% Excessive use: 10% None: 54% With cancer Within recommendation limits 20% Excessive use: 13% None: 51%		
**Ethnicity**	54% of 204 patients with Dutch ethnicity had a cancer diagnose and 50% of 14 patients with another ethnicity		93% of the population from Denmark was referred with NSSC, 4% from another western country, 2% from Middle East/Asia an 1% from other countries 95% of the population from Denmark was diagnosed with cancer, 3% from another western country, 1% from Middle East/Asia an 1% from other countries				94% of patients referred with NSSC were born Sweden and 5% were not born in Sweden	Without cancer: White: 42% Mixed 1% Asian: 6% Black: 20% Other 18% Missing: 31% With cancer White: 56% Mixed 0% Asian: 4% Black: 6% Other 3% Missing: 30%		
**Mental characteristics**										
**Mental diagnosis**		2% of patients with NSSC had dementia and 0% acquired a cancer diagnose	1% was diagnosed after being referred to a Diagnostic Center with a mental or behavioral disorder		7% of referred patients had a mental illness	Moderate to severe mental disorder 5% of patients referred with NSSC	19 (≈7%) of patients referred with NSSC were diagnosed with a mental disorder	Without cancer: 18% With cancer: 13%		
**Mental health related scales**				Health related Quality of life scale: -No difference in physical and social functioning. –Improved emotional function 30 days after referral. -Being unemployed, having 2 or more comorbidities and receiving a cancer diagnosis had the greatest effect. -Gender education and previous cancer did not have any effect on the quality of life.						

* Education: Short < 15 years/skilled worker. Medium academic/trade. Long academic/university level

#### The patient pathway

The patient pathway contains extracted information about the following issues: percentage getting a cancer diagnose, cancer stage, mortality, median time from GP referral to last visit day at a DC or another investigation department, duration of symptoms and all contacts to GP during the 6 months up to referral.

#### Demographic and physical characteristics

Demographic and physical characteristic contained extracted information about the following issues: age (18 years and up), sex (male/female), comorbidities (diseases included in Charlson comorbidity score and other physical diagnoses) and laboratory tests (what tests were performed and what results were).

After being referred with NSCS for further evaluation, the following were also examined:

- Three most prevalent cancer diagnoses found
- Three most prevalent non-cancer diagnoses found
- Three most common symptoms when referred to DC

To get a clear picture of the most prevalent diagnoses and symptoms found within the different study, the three most prevalent diagnoses and symptoms were extracted from all studies.

#### Mental characteristics

Mental health characteristics were found through mental diagnoses, use of anti-depressive medicine and other drugs used for treatment of mental disorders, self-rated health and scales for anxiety, depression and other mental disorders, e.g., Hospital Anxiety and Depression Scale, HADS, and Major Depression Inventory, MDI [30, 31].

#### Social characteristics

Social characteristics were extracted in a socioeconomic frame as marital status, educational level, occupation, income, geographic place of residence, smoking, alcohol usage, ethnicity and other possible described socioeconomic characteristics.

Educational level was seen as Short (< 15 years/skilled worker) Medium (academic/trade) and Long (academic/university level).

## Results

### Study selection

The literature search identified a total of 2751 publications which included 1394 duplicate publications for a total of 1357 original identified publications. A PRISMA flow diagram of the search is presented in Fig. 1. When applying the inclusion and exclusion criteria, 1303 publications were excluded, and 53 publications remained for full text assessment. Of these, 42 publications were excluded due to not being a cohort or case control study (*n* = 26), further duplications (*n* = 1), being published before 1975 (=2), being wrong setting (=4), wrong outcome (=9). Twelve publications in total were included in the systematic review. Næser published two articles [16, 17] from one study and Moseholm published two articles [25, 26] from another study. These studies were analysed together; thus, the results of these studies are described in this review.

### Assessment of the included studies

#### Methodological quality

In the quality synthesis of the nine publications, information for the *Newcastle–Ottawa Quality Assessment Scale* (NOS) was extracted as shown in Table 2. All studies were cohort studies and were published from 2016 to 2022. The study populations ranged from 290 to 23,934 patients. Analysis of the methodological quality of the included articles was assessed using the NOS methods which categorises studies scoring as low quality (0–5 stars), medium quality, (6–7 stars) and high quality (8–9 stars). All ten studies were considered to be of high quality [10, 12, 16, 17, 21, 24–29].

#### Patient characteristics and patient pathway

Eight included studies provided information about social and mental characteristics of participants [10, 12, 16, 17, 21, 23–27]. One study only included physical characteristics and another included physical characteristics and information about patient pathway [28, 29]. Nine publications held information about physical characteristics [10, 12, 16, 17, 21, 24–28].

Six of the ten included studies were from Denmark [12, 16, 17, 24–28] One was from Sweden, Two from UK and one from the Netherlands [10, 21, 23, 29]. The six studies from Denmark, the one from Sweden and the two from UK investigated patients with non-specific symptoms of cancer who were referred to a DC for further examination whereas the study from the Netherlands investigated GPs’ gut feelings regarding cancer possibility [10, 12, 16, 17, 20, 21, 23–28, 32]. The studies from Denmark were conducted in specific regions of the country except for the study by Moseholm et al. which was a nationwide study [12, 16, 17, 24–28].

#### The study population

The study population in the Netherlands study was defined by persons consulting their GP which led to the GP having any kind of gut feeling of cancer independent of clinical signs and symptoms [23]. The study population of the studies from Denmark, the one from Sweden and the two from UK was defined by patients referred to a DC [10, 12, 16, 17, 21, 23–29].

The population of patients with NSSC were divided into two groups. The first group was made up of all patients referred with NSSC, and the second group was made up of patients diagnosed with cancer after being referred with NSSC.

### Patient pathway and characteristics

#### Patient pathway

##### A. Patients referred with NSSC

Between 11 and 35% of all persons referred to a DC or who triggered GP gut feeling were later diagnosed with a cancer [10, 12, 16, 17, 21, 23–29].

The median number of days from referral to last visit day in DC was assessed in two studies from Denmark and ranged from 7 to 10 days [12, 16, 17, 24]. The Swedish study found that the median timeframe from patients first contact in primary care to diagnosis after being referred with NSSC was 37 days [10]. It was also revealed that 77% of patients referred with NSSC were investigated in the DC within 22 days [10]. One study found that the median time interval in primary care for patients diagnosed with cancer was 15 days [27].

Only one study investigated duration of symptoms before patients were referred with NSSC [25, 26]. The median duration of symptoms was 12 days in this study [25, 26]. No studies investigated visits to GP until referral for NSSC. The study from the Netherlands described visits to GP as triggers for referral but no further details were given [23].

##### B. the patients diagnosed with cancer after being referred with NSSC

The 1-year mortality was between 28 and 44% for patients with a cancer diagnosed compared to 2–3% for patients who were not diagnosed with cancer after examination at DC [12, 16, 17]. One study showed that the median survival time after cancer diagnosis was 1,4 years [10].

Two studies examined the stage of cancer and one showed that 47% of patients who attained a cancer diagnose after being referred with NSSC had solid tumors with potential to spread based on TNM-staging [10, 29]. 20% who attained a cancer diagnosis were referred to palliative care [10]. The second study showed how many percent were in the different stages for each cancer diagnose [29]. Four percent of patients with upper Gastrointestinal (GI) cancer, 26% with lung cancer 20% with hematological cancer and 1 % with lower GI cancer were in stage one, 57% of patients with upper Gastrointestinal (GI) cancer, 53% with lung cancer 27% with hematological cancer and 48% with lower GI cancer were in stage one [29].

#### Patient characteristics

##### A. Patients referred with NSSC

Mean age of the included patients was 62–72 years [10, 12, 16, 17, 21, 24–29]. 47–56% of patients referered with NSSC were women and 44–53% were men [10, 12, 16, 17, 23–27, 29]. Four out of seven studies showed hematological cancers as the most frequent cancer diagnosed when referred with NSSC (14–30%) [10, 12, 16, 17, 25, 26, 29]. One study showed that breast cancer (18%) was the most frequent diagnosis while another lung cancer (18%) and a third upper gastrointestinal cancer (22%) as being most frequent [24, 27, 29]. Moreover, the second most prevalent cancers included gastrointestinal cancers (13–23%) in three studies, lung cancers (13–22%) also in three studies and hematological cancers (15%) in one study [10, 12, 16, 17, 24–27, 29]. The third most prevalent cancers were malignant melanoma and hematological, lung, gastrointestinal and kidney cancers [10, 12, 16, 17, 24–27, 29]. Four studies included description of non-malignant diseases diagnosed after referral with NSSC, and three of these four studies showed rheumatological diseases or musculoskeletal disorders as the most common non-malignant diseases found with a diagnostic rate of 5–38% [10, 12, 16, 17, 24]. All four studies showed gastrointestinal diseases as the second most common non-cancerous disease with a diagnostic rate of 7–31% [10, 12, 16, 17, 24].

Eight out of ten studies showed the most frequent symptoms for patients referred with NSSC [10, 12, 16, 17, 21, 23, 25–27, 29]. Weight loss was distinctively the most common symptom for referral with NSSC in seven out of eight studies and presented in 24–66% of patients [10, 12, 16, 17, 21, 23, 25–27, 29]. Fatigue was described as the first, second and third most common symptom and was seen in up to 74% of patients [10, 12, 16, 17, 23, 25–27]. Pain and loss of appetite were also some of the most frequent symptoms seen in patients with non-specific symptoms of cancer [10, 12, 16, 17, 21, 23, 25–27, 29]. Four studies in this review described known comorbidity; hence cardiovascular diseases, lung diseases, diabetes and previous diagnosed cancer were among the most common comorbidities among patients with NSSC [12, 16, 17, 24, 27]. Two studies showed that previously diagnosed cancer, cardiovascular diseases, cerebrovascular disease and diabetes were among the most common comorbidities for patients with NSSC who received a cancer diagnose [12, 24].

##### Mental health

Limited information about mental disorders was described in the studies. One study showed that 7% of the population had a mental illness diagnose when referred with non-specific symptoms of cancer [16, 17]. The same study showed that 2% of the population was diagnosed with a psychiatric disease after being referred with NSSC. Another study showed that 1% of the population was diagnosed with a psychiatric disorder and not cancer after being referred with NSSC [24]. One study showed that 18% of the patientend not diagnosed with cancer had a mental health illness and 13% not diagnosed with cancer had a mental health illness [21]. In one study, 2% of the population had dementia [12]. One study showed that 5% of patients referred with NSSC had mild to moderate mental disorders [27]. None of these patients were diagnosed with cancer. Another study showed that 7% were diagnosed with a mental disorder [10]. No studies reviewed described use of drugs against medical disorders.

##### Socioeconomic factors

Three studies showed that 54–68% of patients referred with NSSC were married/living with a partner and 31–32% were single/widowed/separated [10, 12, 16, 17, 24–26]. Two studies found that 28–37% of patients were employed when referred with NSSC, 1–7% were unemployed and 59–68% were retired [24–26].

One study showed that 81% of the population did not consume alcohol on daily basis and another showed that 10% of patients had a weekly consumption of alcohol above national guidelines [12, 16, 17, 33]. Two studies revealed that 34–38% of patients referred with NSSC never smoked while 61–70% were former or current smokers [12, 16, 17, 25, 26].

##### B. Patients diagnosed with cancer after being referred with NSSC

Four studies showed that 40–58% of patients diagnosed with cancer were women and 42–60% men [12, 21, 24, 28]. Two studies study found that patients diagnosed with cancer had a significant higher age than those not diagnosed with cancer [21, 27].

Ingeman ML et. al [27]. One study found that cancer were more often found in men than women, and another study found that being women were significant associated with getting a cancer [24, 27]. Two studies calculated the odds of getting a cancer diagnose with 1 year increase in age [23, 24]. One found that the odds increased by a factor 1.02% and the other one found that the odds increased by 4% [23, 24].

The percentage of patients who attained a cancer diagnose with medium academic education was 47–52%, with long academic education 6–18% and with short/no education 36–39% [16, 17, 24]. Two studies showed that 56–59% of patients diagnosed with cancer after being referred with NSSC were married patients [16, 17, 24] 17–18% of patients diagnosed with cancer were employed, 0–1% were unemployed and 80–83% were retired [16, 17, 24–26].

One study contained information about the income of the participants [16, 17]. This study showed that 27% of patients who acquired a cancer diagnose were in the lowest income range, 54% in the middle and 19% in the highest [16, 17].

In the Dutch study, 54% of 204 patients with Dutch ethnicity had a cancer diagnose and 50% of 14 patients with another ethnicity [23]. One of the Danish studies showed that 95% of patients with cancer were from Denmark, 3% from another Western country, 1% from Middle East/Asia and 1% from other countries [24]. The English study showed that 56% were white, 6% Black, 4% Asian, and 3% other [21].

Between 36 to 70% of former/current smokers who were referred with NSSC were later diagnosed with a cancer [12, 21, 23]. 20% drank alcohol within recommendation limit, 13% had an excessive use and 51% did not drink alcohol [12, 21].

## Discussion

### Main findings

Up to about one third of patients with non-specific symptoms were diagnosed with cancer after being referred to DC [10, 12, 16, 17, 21, 23–25, 27]. The most common cancer diagnoses were hematological, gastrointestinal and lung cancers, and the most common non-malignant diseases were diagnosed after referral to a DC for rheumatological and gastrointestinal diseases [10, 12, 16, 17, 21, 24–29]. The most common symptoms that triggered a referral were weight loss, fatigue and pain [10, 12, 16, 17, 21, 23, 25–27, 29]. Patients referred with NSSC had a mean age of 64–70 years which was similar to the general world population where the frequency of cancers diagnose gets higher with a higher age [10, 12, 16, 17, 21, 24–29, 34, 35]. Fewer patients who came from a background with higher academic education had a cancer diagnose [10, 16, 17, 24–26]. Seven percent of patients referred with NSSC had a mental diagnose which was less than the background population in Denmark, that has risen from 11,1% in 2014 to 14,2% in 2018 [10, 36].

### Strength and limitations

Our study was conducted and reported according to the PRISMA guidelines to ensure transparency. Despite a thorough literature search, only ten studies were included and seven of these were from Denmark or Sweeden which are a Scandinavian welfare state and may not represent the population or health systems elsewhere [10, 12, 16, 17, 21, 23–29]. Focus on DCs and patients with non-specific symptoms of cancer is a relatively new research area and is reflected by the fact that the oldest study included was published in 2015 [10, 12, 16, 17, 21, 23–29].

Study populations were diverse, and limited patient characteristics were reported in the different studies. Only few patient characteristics were analyzed and presented in a comparable way across the studies [10, 12, 16, 17, 21, 23–29].

All nine studies included in this review were analyzed with good methodological quality assessed by the quality assessment scale NOS [10, 12, 16, 17, 21, 23–29].

### Differences in the studies

The studies from Denmark Sweden and UK investigated patients with non-specific symptoms of cancer who were referred to a DC for further examination for cancer [10, 12, 16, 17, 21, 23–27, 29]. The study from the Netherlands investigated GPs’ gut feeling for cancer independent of clinical signs and symptoms; thus, red flag patients and patients with clear organ specific symptoms could be included in the Dutch study [23]. One Danish study population represented the whole country while the rest represented three different regions in Denmark [10, 12, 16, 17, 24–28]. Currently, CCPs for all organ systems have only been implemented in some European countries [7–11]. Denmark was among the first countries in Europe to introduce Diagnostic Centres for patients referred from their GPs with NSSC which might explain why the vast majority of the studies identified in this review were from Denmark [12, 13, 33].

Looking at the difference between the Dutch study population, the English and the Danish study populations, the Dutch study population had a wider inclusion criterion than the Danish studies which could explain the higher percentage of patients diagnosed with cancer [10, 12, 16, 17, 21, 23–29].

#### Patient pathway

##### A. Patients referred with NSSC

Patients referred with organ-specific symptoms in Denmark had a diagnostic rate at 27–30% for cancer [12]. The percentage of the diagnostic rate was lower in the Scandinavian studies for patients with NSSC, even though patients with previously diagnosed cancer was one of the most prevalent comorbidities [10, 12, 16, 17, 24–28]. This may be attributed to the higher possibility of a non-specific symptom being another disease rather than specific cancer symptoms which makes the hit rate for cancer lower for patients with non-specific symptoms of cancer.

18% of patients with cancer in Denmark do not survive 1 year after getting the diagnosis which is less for patients with NSSC who are diagnosed with cancer [37]. This might indicate that patients with NSSC are diagnosed at a higher cancer stage than cancers presenting specific symptoms of cancer.

Two of the Danish studies showed a median duration time from referral to last visit day was 7 to 10 days which is within the recommended time frame of 22 days [16, 17, 24]. One study showed that the median time duration from first to last visit day was 9 days [12]. The Swedish study found that 77% of patients referred to a DC were investigated within 22 days, and 51% had a time frame from first contact in primary care to diagnosis within 37 days [10].

The short timeframe from GP referral to the last visit day at the DC indicates an effective diagnostic route enhancing a fast diagnostic pathway.

Only one study investigated that the median duration time of symptoms was 12 weeks which reveals that patients with NSSC have symptoms over a longer period before awareness and suspicion of cancer arises [25, 26].

##### B. the patients diagnosed with cancer after being referred with NSSC

Almost half of patients with NSSC who were diagnosed with cancer had a solid tumor with potential to spread based on TNM-staging and 20% went to palliative care according to one of the included studies [10]. This shows that even though patients only have non-specific symptoms, the cancer may be in a well-developed stage and it is therefore important to further examine what other symptoms patients with NSSC present with. Doing this will give a better picture of patients with NSSC which can be used as an indicating tool for GPs to recognize possible cancer patients.

#### Patient characteristics

##### A. Patients referred with NSSC

The most frequent cancer diagnoses found were hematological cancers which are not among the 5 most frequent cancers in the background population [7, 10, 12, 16, 17, 24–27, 29]. Hematological cancers are not characterised by organ-specific symptoms like other cancers are [38, 39]. Thus, hematological cancers are more likely to be referred through a cancer pathway of non-specific symptoms than other cancers that presents with more organ-specific symptoms. Lung and gastrointestinal cancers were the second and third most frequently found cancers in the studies and were also the second and third most frequently diagnosed cancers in the background population [7, 10, 12, 16, 17, 24–27, 29].

Involuntary weight loss was the most frequent symptom associated with cancer in this review and could be caused by other reasons besides cancer (i.e. psychological diseases, gastrointestinal diseases, lung and heart diseases, infections, medicaments, high age etc.) [10, 12, 16, 17, 21, 23–27, 29]. 10–20% of patients who present with weight loss do so for unknown reasons [40, 41]. The frequent unknown reasons for weight loss were also seen with the symptoms of fatigue and appetite loss. The most frequent non-cancerous diseases diagnosed in CCP were rheumatological diseases [10, 12, 16, 17, 24]. These diseases were correlated with fatigue being identified as the common symptom for rheumatologic patients. Fatigue was also one of the most common symptoms that caused GPs to refer patients with NSSC [10, 12, 16, 17, 21 23, 25–27, 29, 42].

The most prevalent comorbidities for patients who acquired a cancer diagnose was previous diagnosed cancer, cardiovascular diseases and diabetes [12, 16, 17, 24, 27]. Cardiovascular diseases and diabetes are some of the most prevalent diseases in the Danish population and are particularly seen in the elderly population [43]. This group was the main population in the studies [10, 12, 16, 17, 21, 23–29]. Previous diagnoses of cancer were also found in some of the studies to be in the top three comorbidities when being  referred with NSSC [12, 24]. Being previously diagnosed with cancer before referral could indicate that GPs are more alert to non-specific symptoms earlier when a patient has had cancer due to the knowledge of an increased risk of second cancer or recurrence of cancer within patients [44]. Patients who have previously had cancer play an important role in this patient group and fast track pathway, and the fast track route may be a faster, easier route for GPs to refer patients compared to other possibilities of diagnoses.

A Danish study from 2008 by Dalton et al. shows that lower social classes has poorer prognosis of survival than higher social classes which may point to prevention and diagnostic strategies aimed at preventing and diagnosing early stage cancer still face challenges and are affected by social inequality [45].

Studies from 2017 and 2019 by Merrild et al. shows that the reason for this might be that people from lower social classes perceive and react differently towards symptoms of diseases such as cancer [46, 47]. Thus, it might not be optimal to use the same signs and symptoms of cancer for this group as in higher social classes [46, 47].

This review displayed that about half of patients with a medium level of education referred with NSSC received a cancer diagnose [16, 17, 24]. This is an aspect that correlates with the abovementioned study from 2008 by Dalton et al. which indicates that cancer strategies have more impact on higher social classes. This contrasts with two studies that found that only 6–14% of those who acquired a cancer diagnose after being referred with NSSC had a higher education [16, 17]. Future analyses of educational level in patients with NSSC would give a better understanding of how socioeconomic inequality impacts the pathway for patients with NSSC. In Denmark, the lower social classes have a high percentage of unemployment [48]. In 2019, two Danish anthropologists examined patients diagnosed with cancer from a low socioeconomic population [47]. These researchers noted that this group of patients “*does not fit into the profile of being proactive healthcare seekers*” [47] even though they have had several contacts to the healthcare system due to comorbidity [47]. It might be that this group of people do not present their symptoms and concern to the physicians and hence have lower probability of being referred through the CCP [46, 47].

Three studies in this review indicated that more than half of the patients with NSSC were married and two studies showed that more than half of the married patients acquired a cancer diagnose [10, 16, 17, 24, 25]. A study from 2008 by Dalton et al. showed that being married resulted in a higher probability of being diagnosed at an early stage compared to unmarried people [45]. Assuming that married people are more likely go to the doctor when they experience non-specific symptoms compared to unmarried people, this may support the argument that married people get an earlier stage diagnose compared to unmarried people [45]. The result of the current review indicates that the same assumption could be drawn about patients with NSSC and shows that marital status might be a factor in how patients obtain access to the system.

A Danish analysis from 2021 shows that patients with mental diseases are represented unequally within social parameters compared to the general population [23]. More people living alone and more people having the primary school, as the highest education completed, had a mental diagnose compared to not living alone and not having primary school as the highest education level [23]. The analysis also showed that people with mental diagnoses had more contacts to the health system than the general population [23]. The current review found that only 7 % of patients referred with NSSC had a mental diagnose, which is lower than for the general population in Denmark [10, 36, 49]. This might indicate that patients with mental diagnoses also does not present their symptoms and concern to the physicians or that the physicians do not capture the signs and symptoms of NSSC that these patients show.

##### B. the patients diagnosed with cancer after being referred with NSSC

This review found that most of the population was retired and the rest were employed when referred with NCCS [16, 17, 24–26]. The risk of getting cancer rises with age which might explain the high number of retired patients in this review. A more illustrative measure of social inequality in this type of patient group might therefore examine what branch of work these patients have/had as well as their income and highest education levels.

In this review, one study found that 27% of patients with NSSC who received a cancer diagnose had low, 54% had middle and 19% high income [16, 17]. These results may indicate that a smaller number of patients with low income were referred with NSSC compared to patients with medium or high income. Furthermore, it could indicate that the low social classes do not express themselves in the same way as patients in the middle and higher social classes.

## Conclusion

Overall, limited information was found on patient pathway and characteristics of patients with NSSC. The limited number of studies found in this review did not include enough heterogenetic data to perform a meta-analysis.

The most common diagnoses, symptoms and comorbidities of patients referred with NSSC were described in most studies and with most heterogenic data. The symptoms present when patients referred with NSSC correlates with the most frequent malignant and non-malignant diagnoses given. One specific patient group emerged in this review. We found that if patients had previous cancer, it could be a trigger for the GP to refer the patient with NSSC through the cancer package pathway. Previous cancers were one of the most frequent comorbidities for NSSC patients receiving a cancer diagnose.

It is still unclear if mental or socioeconomic characteristics trigger GPs to suspect NSSC in patients and what the influences of these characteristics are on patient pathways.

Further studies concerning socioeconomic, mental and physical characteristics as well as patient pathway is necessary to assess enough data for a meta-analysis. This could improve our understanding of the characteristic picture and its influence on the patient pathway through the system. Potentially, this information could give GPs a better understanding of which patient characteristics should trigger a concern on NSSC and thereby be able to recognize a cancer diagnose at an earlier stage.

## Data Availability

All data generated or analysed during this study are included in this published article.
